# A Clinical Care Algorithm for Detecting Progression in Multiple Sclerosis: RetratEMos Project

**DOI:** 10.7759/cureus.74001

**Published:** 2024-11-19

**Authors:** José E Meca-Lallana, René Robles, Lamberto Landete, Nieves Téllez, José M García-Domínguez, Pilar Garcés, Lucienne Costa-Frossard

**Affiliations:** 1 Clinical Neuroimmunology Unit, Neurology Department, “Virgen de la Arrixaca” Clinical University Hospital (IMIB-Arrixaca), Murcia, ESP; 2 Multiple Sclerosis and Clinical Neuroimmunology, NICEM Cathedral, UCAM-San Antonio Catholic University, Murcia, ESP; 3 Neurology, Hospital Universitario Dr. Josep Trueta, Girona, ESP; 4 Neurology, Hospital Universitario Doctor Peset, Valencia, ESP; 5 Neurology, Hospital Clínico Universitario de Valladolid, Valladolid, ESP; 6 Neurology, Hospital General Universitario Gregorio Marañón, Madrid, ESP; 7 Medical Department, Novartis Farmacéutica, S.A., Madrid, ESP; 8 Neurology, Hospital Ramón y Cajal, Madrid, ESP

**Keywords:** algorithm, clinical practice, multiple sclerosis, progression, secondary progressive multiple sclerosis

## Abstract

Objective: The diagnosis of secondary progressive multiple sclerosis (SPMS) is often established retrospectively leading to a delay in detection. This work presents a clinical care algorithm that aims to facilitate the recognition of the secondary progressive phase of the disease, analyzing its usefulness and the feasibility of its implementation in routine clinical practice.

Methods: The algorithm was developed in four phases: 1) choice of validated diagnostic tools for the detection of progression; 2) assessment of these tools based on experience of use, applicability, time consumed, perceived usefulness and suitability for a profile of a patient in transition to SPMS; 3) framework and final sequence of application; 4) feasibility evaluation through application in clinical practice.

Results: A hierarchical algorithm was developed with an initial screening phase to detect warning signs and establish suspicion of progression (which included the tests "Your Multiple Sclerosis (Your MS)," "MSProDiscuss," and "Nomogram") and a second phase conditional on a positive result in the first, including a functional examination with the Symbol Digit Modalities Test (SDMT), 9-Hole Peg Test (9-HPT), and Timed 25-Foot Walk (T25FW) tools. The algorithm was applied to 373 patients with Expanded Disability Status Scale (EDSS) ≥ 2. The mean time spent per patient in the screening was eight minutes and 20.4 minutes for the complete algorithm. The perceived usefulness of the process by the neurologists was 3.1 (range of 1-4, with 4 being the maximum). In 46% of the cases, the algorithm detected the need for additional functional exploration.

Conclusions: From our experience, this clinical care algorithm is effective and feasible for detecting progression in MS, although its implementation requires proper organization and can be uneven depending on the resources of each center.

## Introduction

According to the third edition of the Multiple Sclerosis Atlas, the estimated number of people affected by multiple sclerosis (MS) worldwide in 2023 was 2.9 million. MS is the leading cause of non-traumatic neurological disability in young adults in the Western world [1], and in Spain, recent studies indicate an increasing prevalence, with figures ranging from 100 to 143 cases/100,000 inhabitants [2,3] and an estimated number of more than 50,000 people affected.

The most common initial form (85-90% of cases) is the relapsing-remitting phenotype (RRMS), characterized by episodes of neurological deficit (relapses) with complete or partial recovery in between. Natural history studies show that approximately 50% of patients who start with RRMS will progress to secondary progressive MS (SPMS) within 15-20 years [4-7]. Conversion to SPMS entails a gradual and irreversible accumulation of disability independent of relapses, as a result of chronic inflammation, demyelination, and oxidative stress, among other neurodegenerative phenomena [8].

However, nowadays, the diagnosis of SPMS is complex and, in most cases, made retrospectively, when the patient already has a high and irreversible degree of disability [9]. The disparity of diagnostic criteria and the lack of biomarkers that define the transition to the progressive phase of the disease can lead to a delay in diagnosis of up to three years [10]. Recognition of the transition phase to SPMS is often based on patients’ perceptions of subtle changes in their symptoms that have a functional impact on their daily life [11]. Neurologists, in turn, may be reluctant to make a definitive diagnosis of the progressive phase of the disease because of limited treatment options or difficulties in communicating the diagnosis [12].

There are currently models based on algorithms and nomograms such as the MS Prediction Score [13], the SMPS nomogram (hereinafter Nomogram) [14], and the Lorscheider calculator [15] that can estimate the risk of progression to SP form, although the factors identified as predictors of conversion are not consistent across studies [16]. The MSProDiscuss tool facilitates physician-patient discussion of disease progression in clinical practice [16-21].

These works reflect the increasing efforts being made to detect the transition to a progressive course. Following this line, a group of neurologists with extensive experience in the management of MS, and aware of the implications of a delay in identifying the transition to a secondary progressive phase in the clinical evolution of these patients, have developed a clinical care algorithm to facilitate the early detection of progression by integrating recognized tools used in daily practice for the detection of progression in MS.

The aim of this work was to describe the characteristics, advantages, and limitations of this algorithm as a pathway for the early detection of progression to SPMS and to evaluate its feasibility and usefulness in order to consider its application in clinical practice. The analysis of its predictive capacity for the diagnosis of SPMS is not part of this first approach.

This article was previously presented as a meeting abstract at the Annual Meeting of the Spanish Society of Neurology (SEN) in May 2024.

## Materials and methods

Development of the clinical care algorithm

First, a collaborative working group of six Spanish neurologists specialized in the care and treatment of people with MS was formed. Their selection ensured national representation of different types of centers. The working group was responsible for developing the algorithm and assessing its feasibility. This study did not involve patients, and therefore no patient data were collected. Experts provided their opinion on the feasibility and usefulness of the algorithm for detecting suspected early progression of MS, from a clinical perspective via an online questionnaire, without involving any patient data.

The algorithm was developed in three phases between January and July 2021.

First Phase

Selection of the tools to be included in the algorithm. Based on their clinical experience and a review of the literature, the working group selected the diagnostic tools to be included: Your MS, MSProDiscuss [16-21], Nomogram [14], EDSS (Expanded Disability Status Scale), 9-HPT (Nine-Hole Peg Test) [22], SDMT (Symbol Digit Modalities Test) [23], EDSS-Plus [24] (EDSS + T25FW (Timed 25-Foot Walk) + 9-HPT), and Prediction Score Calculator [13] (Table 1). 

**Table 1:** Selected diagnostic tools to be included in the algorithm * During the development of this manuscript, the website for accessing MSProdiscuss was no longer available. For more information on the release of the MSProDiscuss tool, please contact medical.info@novartis.com. 9-HPT, Nine-Hole Peg Test; EDSS, Expanded Disability Status Scale; MS, multiple sclerosis; MS, multiple sclerosis; SDMT, Symbol Digit Modalities Test; SPMS, secondary progressive multiple sclerosis; T25FW, timed 25-foot walk

Tool	Features
Your MS [25] (https://es.ms.your-symptom-questionnaire.com/)	A questionnaire addressed to the patient with questions about his or her MS in the last 6 months. Includes information on outbreaks, symptoms and impact on daily life.
MSProDiscuss* [16-21]	A validated clinician support tool for the study of MS progression. It asks about the symptoms and sequelae experienced by the patient in the last 6 months and translates them into a color code in reference to the risk of progression.
Nomogram [14,26] (https://aliman.shinyapps.io/SPMSnom/)	A tool with predictive capacity for conversion to SPMS at 10, 15, and 20 years according to the following factors: year of birth, age at diagnosis, sex, first recorded EDSS and age at first recorded EDSS.
EDSS	It evaluates the degree of disability in 8 functional systems (pyramidal, cerebellar, brainstem, mental, sensory, visual, bowel and bladder). A score > 4 primarily defines the patient's difficulty in walking.
EDSS-Plus [24]	A composite endpoint adding the T25FW and 9-Hole Peg Test to EDSS for SPMS disability progression assessment.
9-HPT [22]	A brief, quantitative test of manual dexterity and function impairment. It consists of placing nine sticks in their corresponding holes and then removing them as quickly as possible. The time taken by the patient to insert and remove the sticks is recorded.
SDMT [23]	A test for the rapid detection of cognitive dysfunction by means of a classic task of substituting symbols for digits from an established key. In MS it is one of the reference tests to evaluate cognitive symptoms.
MS Prediction Score Calculator [13]	A calculator that estimates the current individual risk of transition to PMSS based on the patient's current age, time since last flare, and the tract(s) affected by the current main symptoms (optic, sensory, vestibular, motor, undefined), and whether there has been complete remission of the last flare.

Second Phase

The working group evaluated each tool in terms of experience of use, applicability, burden of time consumption, perceived usefulness, and suitability for a patient profile in transition to secondary progression. Using an electronic questionnaire, they were asked to rate each of these aspects on a numerical scale from 1 to 4 (where 1 is "minimum" and 4 is "maximum," i.e., maximum applicability).

Third Phase

The results of the first two phases were analyzed by the working group in an online meeting, where the following aspects were systematically addressed: 1) identification of the patient profile to be used; 2) physician's subjective assessment of the ability of the tools to detect early signs of progression and feasibility of use in their practice; 3) analysis of signs or symptoms that, in the experience of the members of the working group, may indicate a possible progression (hereinafter referred to as “warning signs”); 4) final selection of the tools to be included into the algorithm based on the score obtained; and 5) tool application sequence.

Application and assessment of the algorithm

Once the algorithm was developed, each member of the working group used it in his/her clinical practice for three months on a target sample of at least 60 people with MS to assess its applicability and feasibility. Given the lower prevalence of SPMS at low EDSS scores [27], it was agreed to apply the algorithm to patients with an EDSS score ≥2.

While the algorithm was being applied, each neurologist completed an electronic questionnaire for each patient, recording (using a numerical scale from 1 (minimum) to 4 (maximum)) the difficulty of using each tool, the time taken to apply it, and the usefulness of the information provided in identifying suspected progression. The time taken to complete the process and the perceived usefulness were recorded. In addition to the weighting, the specialists were asked to indicate any observations they had noticed when using the application in order to improve its understanding and management in practice. As this was a study of usability and applicability, the questionnaire did not require the collection of the final score for each of the tools.

Statistical analysis

Due to the small sample size of participating neurologists (n = 6), no formal statistical analysis was performed. The scores from the numerical scales used by the working group to rate the different aspects of the algorithm process are presented as means.

## Results

Algorithm development

**Figure 1:**
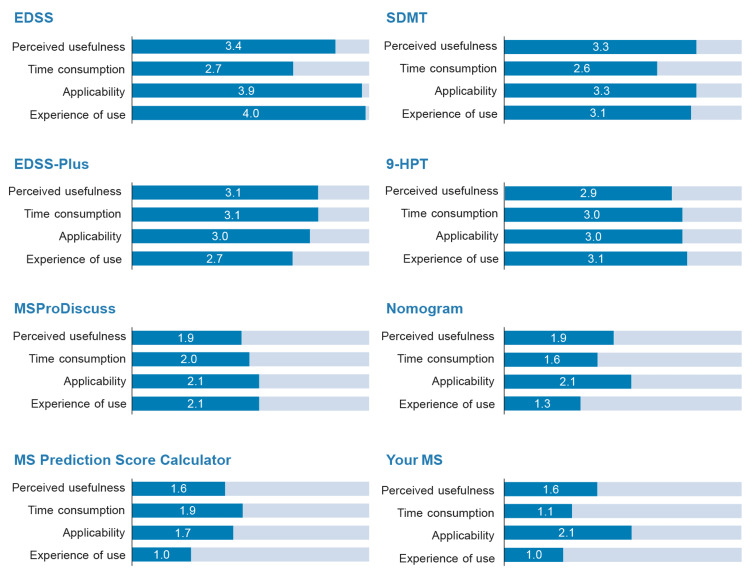
Usefulness, applicability, and experience of use of the standard tools in the detection of progression in MS. The data represent the mean of the scores given by the working group. The score range of the numerical scales used to rate all aspects assessed was 0 to 4, with 1 being "minimum" and 4 being "maximum." EDSS plus is a composite endpoint adding the T25FW and 9-HPT to EDSS for disability progression assessment. 9-HPT, Nine-Hole Peg Test; EDSS, Expanded Disability Status Scale; MS, multiple sclerosis; SDMT, Symbol Digit Modalities Test; T25FW, timed 25-foot walk

In the first phase, it was observed that the EDSS scale scored highest in terms of experience of use (4.0), applicability (3.9), perceived usefulness (3.4), and burden of time consumption (2.7), followed by the SDMT and 9-HPT (Figure 1).

The complementary information that the neurologists felt the other tools added to the EDSS scale is shown in Table 2. 

**Table 2:** Complementary information that neurologists believe the assessed tools contribute to EDSS * During the development of this manuscript, the website for accessing the MSProDiscuss was no longer available. For more information on the release of the MSProDiscuss tool, please contact: medical.info@novartis.com. 9-HPT, Nine-Hole Peg Test; EDSS, Expanded Disability Status Scale; MS, multiple sclerosis; SDMT, Symbol Digit Modalities Test; SP, secondary progressive

Tool	Additional information to the EDSS
Nomogram [14,26] (https://aliman.shinyapps.io/SPMSnom/)	It allows a baseline picture of the patient to be taken and the long-term prognosis of progression to a SP form to be estimated. However, the variables that make up the scale (year of birth, age at onset, sex, first EDSS score, age at first EDSS) are already collected in the clinical history. It could be useful to draw the attention of less experienced specialists.
MSProDiscuss* [16,18-21]	It allows to identify the moment of the evolution of the disease. It objectifies what is intuitively suspected (even without EDSS variation) and can assist the neurologist in communicating with the patient. Useful to systematically review functional systems and make the patient aware of possible worsening. It would be interesting to perform it in all patients in order to perform a longitudinal analysis. It will soon be available as a diagnostic tool in a mobile application.
Your MS [25] (https://es.ms.your-symptom-questionnaire.com/)	It is a useful tool that helps to raise suspicions. Systematizes the collection of the usual anamnesis variables. Anticipate to the patient the issues to be addressed in the consultation. It should be used longitudinally.
9-HPT [22]	It provides a lot of additional information on upper extremities. Results can be influenced by fatigue, temperature, and time of day. It should be performed annually on all patients.
SDMT [23]	It is useful and fast. Good screening tool for cognitive impairment to be performed without suspicion of progression. A snapshot of cognitive status is obtained but the large intraindividual variability requires confirmation at 3-6 months. As a negative aspect, the learning of the test by the patient stands out.
EDSS-Plus [24]	It is useful for measuring response to treatment in progressive forms. Difficulty for systematic administration due to lack of time. To be performed only if SP is suspected.
MS Prediction Score Calculator [13]	Unknown

Participating neurologists identified new symptoms that had not been reported by patients in previous consultations, mainly fatigue, subjective cognitive impairment, spasticity, lower extremity pain, loss of balance, gait slowing, frequent stumbling, and falling to the floor, as important signs to suspect possible progression.

The experts agreed that the most useful tools for early detection of progression were the functional tests of cognitive processing speed (SDMT) and manual dexterity (9-HPT). Taking into account their experience with the "EDSS-Plus scale" [24], it was decided to also include the T25FW for gait assessment in the algorithm. However, given the lack of time and resources to systematically, routinely, and jointly administer these three functional tests to all patients seen in the physician's office, it was agreed that "Your MS," "MSProDiscuss," and "Nomogram" would be the first tools to be applied as part of the care algorithm, to detect warning signs of progression and identify those patients who need further functional assessment with the SDMT, 9-HPT, and T25FW tests. According to the participants, "Your MS" allows bidirectional feedback with the patient and "MSProDiscuss" explores multiple aspects of the patient's lifestyle. The Nomogram, although the least used tool, was highly valued by some experts for initial screening because of its ability to estimate the probability of reaching SPMS during the course of the disease. Finally, the algorithm was structured in such a way that the detection of warning signs in any of the screening tools ("Your MS," "MSProDiscuss," "Nomogram," or anamnesis), would imply an additional functional examination with SDMT, 9-HPT, and T25FW. The EDSS and EDSS-Plus were not included in the algorithm, as only an increase in their score is definitive for SPMS.

**Figure 2:**
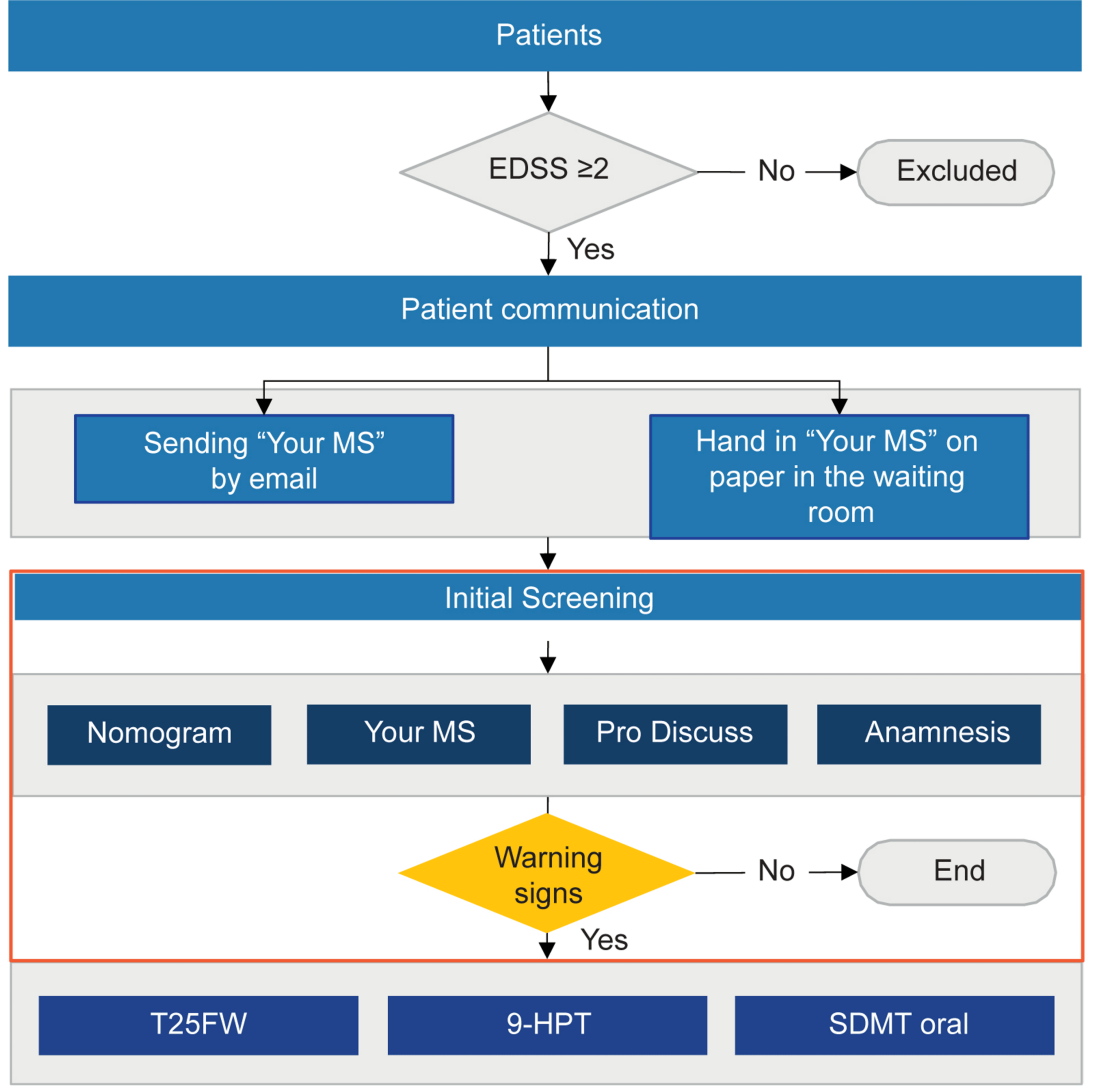
Care process algorithm for the early detection of signs of progression in multiple sclerosis 9-HPT, Nine-Hole Peg Test; EDSS, Expanded Disability Status Scale; MS, multiple sclerosis; SDMT, Symbol Digit Modalities Test; T25FW, Timed 25-Foot Walk

The warning signs to be detected during the screening phase were not defined quantitatively but qualitatively. These were considered to be the presence of a yellow or red result in "Pro-Discuss," an increase in the number of symptoms and the impact of MS on the patient's life in "Your MS," a high score on the Nomogram and subjective complaints of the patient not previously reported in consultation such as spasticity, stumbling when walking, fatigue, or subjective cognitive impairment. The care process algorithm is shown in Figure 2.

Applicability and assessment of the usefulness of the clinical care algorithm

The algorithm was applied to 373 patients. Warning signs were identified in 170 (46%) patients after initial screening, of whom 150 (88%) underwent SDMT, 9-HPT, and T25FW scans. Twenty patients were unable to complete the process in a single visit.

The mean application time for the complete algorithm was 20.4 minutes, corresponding to a mean application time for the Nomogram of 2.5 minutes, MSProDiscuss 4.2 minutes, SDMT 5.3 minutes, 9HPT 4.1 minutes, and T25FW 2.9 minutes. The time of administration of "Your MS" was not recorded because the patient completed it while waiting to be seen, so as not to shorten the visit, nor was the time of the anamnesis taken because this is part of standard clinical practice.

The perceived usefulness of the algorithm by the neurologists received a mean score of 3.1 out of 4. The process was considered useful because it allows for the systematic application of standardized tools to detect progression and identify those patients at risk who require more in-depth monitoring at subsequent follow-up visits. Neurologists noted that the application of the care process tools could consume more consultation time and that factors such as age, high EDSS, and time of disease progression had a significant weight in the risk weighting of the initial tests applied for progression. Although signs of progression were detected in some patients with an EDSS score of less than 2.5, the working group agreed that, due to the lower prevalence of progressive forms in patients with lower EDSS scores [15,27], the use of the algorithm in patients with an EDSS score of ≥3 could be useful in centers with limited resources; in fact, neurologists reported great utility in the EDSS range of 3-5.

**Figure 3:**
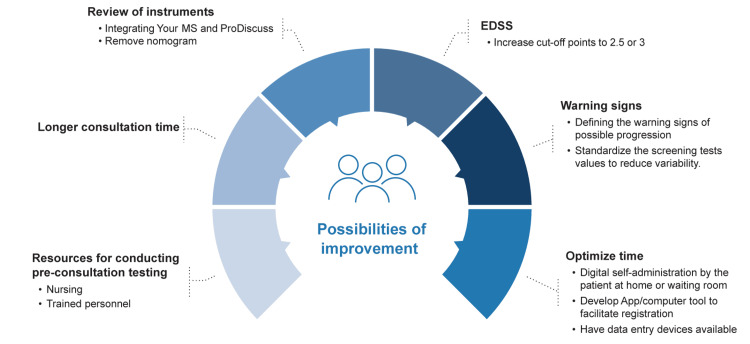
Critical aspects for improvement in the adoption of the process in daily practice. EDSS, Expanded Disability Status Scale; MS, multiple sclerosis Figure created by the authors

Some specialists noted difficulty and high time consumption in applying the Nomogram to patients with a long evolution, where it was difficult to access data from the first examination due to the recent incorporation of electronic medical records in their centers. Figure 3 illustrates the key areas that the working group felt needed improvement in order to successfully integrate the process into clinical practice.

## Discussion

Lack of standardization, low sensitivity of EDSS as the sole diagnostic measure [9,28], and the difficulty in communicating with the patient complicate the early detection of progression in MS. The implementation of an algorithm based on standardized tools during the care process can support neurologists in their clinical practice. It is important to highlight that this work does not analyze the ability of the algorithm to diagnose SPMS, but rather evaluates its implementation and feasibility for detecting or suspecting progression. The lack of time and resources at visits limit the systematic performance of the standard clinical exams, and this pilot study provides guidance for the detection of SPMS and the selection of those cases that require more exhaustive follow-up, as well as evaluating its implementation and feasibility.

The results show that neurologists spent about eight minutes per patient to identify warning signs of possible progression in patients with EDSS ≥2, and that in 46% of cases, the application of screening tools alerted to the need for further exploration with specific tools. The assistance process was generally considered useful or quite useful, as there was a clear need to establish a method, beyond the Lorscheider’s definition [15], that would allow progression to be suspected before being confirmed by an increase in EDSS. By adopting this approach, neurologists would be able to discuss with the patient at an early stage the possibility of transition and a possible change in the course of his or her disease and to anticipate possible changes in the pharmacological and non-pharmacological therapeutic attitude that could improve the patient's quality of life.

Although patients with an EDSS>2 were initially selected to apply the algorithm, it was recognized that the care burden and resource shortages in certain centers could limit its implementation in practice, as this population represents approximately 80% of patients [29]. In addition, the working group felt that having trained personnel to perform the tests prior to the start of the consultation would make its application more efficient in terms of benefits obtained and efforts invested, at the risk of excluding incipient secondary progressive forms.

One of the limitations of the algorithm may be the lack of a quantitative definition of the warning signs of progression in the screening phase, which may introduce some variability in the interpretation of the initial screening, although the aim of the project was to assess the feasibility of applying the algorithm in daily practice. Analysis of its potential diagnostic accuracy and validation could be the focus of future research.

The authors consider it is important to inform that during the development of this manuscript, the website for accessing the MSProDiscuss tool was no longer available. Although this may limit the partial application of the algorithm in the screening phase, there is still the possibility of integrating it into the center's computer system. The care process presented is intended to optimize current clinical practice and should be considered dynamic and flexible, adapting to the specific environment of each center and evolving to other future models that improve the care of MS patients. Therefore, given the possibility of changes in the availability of the tools used over time, it has been considered relevant to share this clinical experience.

## Conclusions

This work shows a clinical care algorithm developed using standardized tools and the experience of its application in practice. The specialists involved found it to be an easy tool to integrate into daily clinical practice to detect suspected early progression in multiple sclerosis. The search for solutions to the challenge of early detection of progression is necessary, especially in centers where sufficient resources are not available. This algorithm proposes the implementation of tools to facilitate physician-patient communication during disease state assessment and transition discussion. It also aims to help identify early signs of progression and provide a window of opportunity for treatment.
